# Public Knowledge and Attitude toward Essential Tremor: A Questionnaire Survey

**DOI:** 10.3389/fneur.2016.00060

**Published:** 2016-04-22

**Authors:** Sherif Shalaby, Jeffrey Indes, Benison Keung, Christopher H. Gottschalk, Duarte Machado, Amar Patel, Daphne Robakis, Elan D. Louis

**Affiliations:** ^1^Department of Neurology, Yale School of Medicine, Yale University, New Haven, CT, USA; ^2^Department of Vascular Surgery, Yale School of Medicine, Yale University, New Haven, CT, USA; ^3^Department of Chronic Disease Epidemiology, Yale School of Public Health, Yale University, New Haven, CT, USA; ^4^Center for Neuroepidemiology and Clinical Neurological Research, Yale School of Medicine, Yale University, New Haven, CT, USA

**Keywords:** essential tremor, knowledge, attitudes, clinical, survey

## Abstract

**Background:**

Public awareness of and attitude toward disease is an important issue for patients. Public awareness of essential tremor (ET) has never been studied.

**Methods:**

We administered a 10-min, 31-item questionnaire to 250 consecutive enrollees. These included three samples carefully chosen to have a potential range of awareness of ET: 100 individuals ascertained from a vascular disease clinic, 100 individuals from a general neurology clinic, and 50 Parkinson’s disease (PD) patients.

**Results:**

Leaving aside PD patients, only 10–15% of enrollees had ever heard of or read about “ET.” Even among PD patients, only 32.7% had ever heard of or read about ET. After providing enrollees with three synonymous terms for ET (“benign tremor,” “kinetic tremor,” “familial tremor”), ~40% of non-PD enrollees and 51.0% with PD had ever heard or read about the condition. Even among participants who had heard of ET, ~10% did not know what the main symptom was, 1/3 were either unsure or thought ET was the same disease as PD, 1/4 thought that ET was the same condition as frailty- or aging-associated tremor, 2/3 attributed it to odd causes (e.g., trauma or alcohol abuse), only 1/3 knew of the existence of therapeutic brain surgery, fewer than 1/2 knew that children could have ET, and 3/4 did not know of a celebrity or historical figure with ET. Hence, lack of knowledge and misconceptions were common.

**Conclusion:**

Public knowledge of the existence and features of ET is overall poor. Greater awareness is important for the ET community.

## Introduction

Essential Tremor (ET) is one of the most common neurological diseases (1–3), occurring in 1 of every 25 people aged 40 years and older (4). It is estimated to affect seven million individuals in the United States alone (5). The tremor is generally progressive (6, 7) and can be debilitating; as 15–25% of patients with ET retire prematurely, and 60% choose not to apply for a job or promotion because of uncontrollable shaking in their hands (8). The shaking is often embarrassing for patients (9), who are concerned about how they will be perceived by others. Social anxiety as well as social phobia can accompany the disorder (10, 11), and some patients are reluctant to be seen in public.

In general, public awareness of and attitude toward disease is an important issue for patients. Misconceptions, negative attitudes, and stigma from disease may have a profound effect on patients’ quality of life (12). For this reason, public knowledge and awareness of neurological disease has been studied extensively; for example, in epilepsy alone, there are more than 20 such studies (12, 13). Despite its high prevalence and potential to produce disability, ET does not garner the same public attention as a related tremor disorder, Parkinson’s disease (PD), which is less prevalent than ET. To our knowledge, public awareness of ET and the accuracy of the public’s knowledge of ET have never been studied.

The goal of the current survey was to determine the extent of awareness of ET and to gauge general knowledge about ET among individuals who are free of ET. To gain additional insights and perspective, we also assessed awareness and knowledge among patients with a related tremor disorder, PD. Finally, we examined whether a range of demographic factors (e.g., age, gender, and education) influenced this knowledge.

## Materials and Methods

### Subjects and Setting

From August 1, 2015 through October 31, 2015, we consecutively enrolled 250 individuals in order to conduct a 10-min in-person interview. As personal medical data were not collected during the interview, the Yale University School of Medicine Internal Review Board determined that signed informed consent was not necessary. By design, three samples of participants were carefully chosen with an expectation that level of awareness of neurological problems, and ET more specifically, might differ subtly across the three. The first sample comprised 100 persons who were expected to have the least knowledge of ET. These were individuals attending a routine outpatient visit at the Yale Vascular Disease Clinic. An accompanying person(s) (e.g., family member) was also interviewed, if available. The patient and accompanying person were interviewed separately so as not to influence one another’s responses. The second sample comprised 100 persons. These were general neurology patients (e.g., headache, low back pain, and neuropathy) and their accompanying person(s), if available, who were attending a routine outpatient visit in the Yale General Neurology Clinic. The patient and accompanying person were interviewed separately. The expectation was that the second sample group might be more aware of ET than the first. The third group comprised 50 PD patients who were attending a routine outpatient visit in the Yale Movement Disorders Clinic. They were expected to have the greatest awareness of ET.

Enrollment during this period was on certain days of the week, depending on the availability of the interviewer (SS). On interview days, the interviewer approached all patients who were seeing one of the participating doctors on that day (JI in the vascular disease clinic, BK and CG in the general neurology clinic, and DM, AP, and DR in the movement disorders clinic). As these were adult clinics, only individuals aged 18 years and older were enrolled.

### Questionnaire

The questionnaire was designed by two of the authors (SS and EL), with one of them (EL) being a senior movement disorders neurologist with a special interest in tremor research. These authors consulted published questionnaires on public knowledge of other neurological disorders [e.g., see Ref. (13)], making modifications and adding items that were more relevant to ET. After piloting the questionnaire on 15 enrollees (not included in our final sample of 250 participants), final modifications were made.

The final questionnaire comprised 31 questions, which included single choice, multiple selection, or fill-in-the-blank responses. The first several questions were demographic (age, gender, education, ethnicity, and occupation). Participants were then asked, “Have you ever heard of or read about a disease called ‘essential tremor’?” Because this question required knowledge of a single term, if the respondent replied “no”, the interviewer then provided three synonymous terms (i.e., “sometimes also known as ‘benign tremor,’ ‘kinetic tremor,’ or ‘familial tremor’”) to see whether this garnered additional positive responses.

We also wanted to exclude participants who themselves might have ET, as their knowledge of ET would skew the results. Hence, we asked a series of five screening questions about ET (presence of uncontrollable shaking or tremor; prior diagnosis of ET; presence of arm tremor; presence of head tremor; and presence of voice tremor). If a non-PD participant answered “Yes” to any one of the five ET screening questions, then the interview was terminated. If a PD participant answered “Yes” to any one of three of the ET screening questions (prior diagnosis of ET; presence of head tremor; presence of voice tremor), the interview was similarly terminated.

Participants (*n* = 99) who (1) answered affirmatively to the initial question about awareness of ET and (2) did not screen positive for ET, were asked to continue, answering the remaining 15 questions that asked about the clinical features, disease course, and treatment of ET.

A 3-min, 5-item mini-mental state screen was also performed to briefly assess attention, orientation, calculation, and recall in PD patients.

### Statistical Analysis

All calculations were performed in SPSS statistical software (version 21; SPSS Inc., Chicago, IL, USA). All tests were two-sided, and significance was accepted at the 5% level. Using analysis of variance (ANOVA) and chi-square tests (or Fisher’s exact test), we compared demographic factors across the three samples of patients. Each of the five subgroups (vascular disease patients, their accompanying persons, general neurology patients, their accompanying persons, and PD patients) was asked, “Have you ever heard of or read about a disease called ‘essential tremor’?” Using a chi-square test (or Fisher’s exact test), we compared the proportion in each subgroup who had responded affirmatively. We compared affirmative responders to those who did not respond affirmatively in terms of demographic factors, using Student’s *t*-tests and chi-square tests. In a multivariate logistic regression model that included age, gender, Caucasian race, educational level (bachelor’s degree or higher vs. other), and occupation (health-related vs. other), we assessed the predictors of affirmative response; these analyses yielded odds ratios (OR) and 95% confidence intervals (CI). We compared 25 PD vs. 74 non-PD participants in terms of their responses to 15 questions about the clinical features, disease course, and treatment of ET.

## Results

There were 250 participants [100 in sample 1 (55 vascular disease patients and 45 accompanying persons), 100 in sample 2 (79 general neurology patients and 21 accompanying persons), and 50 in sample 3 (50 PD patients)] (Table 1). There were no refusals. Thirteen (5.2%) of 250 participants screened positive for ET, including four (1.6%, three general neurology patients and one vascular disease patient) who had been diagnosed with ET previously. These 13 participants were excluded from the analysis; 237 participants were included.

**Table 1:** **Demographic factors of each sample of participants**.

	Sample 1		Sample 2		Sample 3	
	Vascular disease patient	Accompanying vascular disease patient	General neurology patient	Accompanying general neurology patient	PD patient	*p*-value comparing all groups
*n*	55	45	79	21	50	
Age in years	62.5 ± 15.1	60.1 ± 17.9	49.7 ± 16.2	62.1 ± 13.5	68.6 ± 10.9	<0.001^a^
Female gender	27 (49.1)	19 (42.2)	49 (62.0)	14 (66.7)	22 (44.0)	0.09^b^
Caucasian	40 (72.7)	40 (88.9)	65 (82.3)	17 (81.0)	45 (90.0)	0.14^b^
Education						0.16^b^
<High school	7 (12.7)	3 (6.7)	4 (5.1)	0 (0.0)	5 (10.0)
High school	27 (49.1)	20 (44.4)	41 (51.9)	11 (52.4)	19 (38.0)	
Bachelor	12 (21.9)	15 (33.3)	10 (12.7)	4 (19.0)	14 (28.0)	
Masters	8 (14.5)	4 (8.9)	22 (27.8)	5 (23.8)	9 (18.0)	
Doctorate	1 (1.8)	3 (6.7)	2 (2.5)	1 (4.8)	3 (6.0)	
Health-related occupation^c^	8 (14.5)	7 (15.6)	17 (21.5)	4 (19.0)	5 (10.0)	0.51^b^
Screened positive for ET	4 (7.3)	2 (4.4)	6 (7.6)	0 (0.0)	1 (2.0)	0.76^b^

*Number of participants (percentage) or mean ± SD*.

*^a^ANOVA test*.

*^b^Chi-square test or Fisher’s exact test*.

*^c^Physician (*n* = 2), physician assistant (*n* = 2), nurse (*n* = 10), home health aid (*n* = 7), medical research (*n* = 3), and other healthcare occupation (*n* = 12)*.

Each sample was asked, “Have you ever heard of or read about a disease called ‘essential tremor’?” Those who initially responded affirmatively included 8/51 (15.7%) vascular disease patients and 7/43 (16.3%) of their accompanying persons, 6/73 (8.2%) general neurology patients and 2/21 (9.5%) of their accompanying persons, and 16/49 (32.7%) PD patients (chi-square test = 13.71, *p* = 0.008). After the provision of three synonymous terms for ET, affirmative responses (*n* = 99 participants) were as follows: 21/51 (41.2%) vascular disease patients and 17/43 (39.5%) of their accompanying persons, 30/73 (41.1%) general neurology patients and 6/21 (28.6%) of their accompanying persons, and 25/49 (51.0%) PD patients (chi-square test = 3.34, *p* = 0.50).

Demographically, the 99 participants who responded affirmatively were similar in age to the 138 participants who did not (59.0 ± 17.4 vs. 59.2 ± 16.0 years, Student’s *t-*test = 0.11, *p* = 0.91) but they were more likely to be women [59/99 (59.6%) vs. 63/138 (45.7%), chi-square = 4.49, *p* = 0.03] and to have attained a bachelor’s degree or higher [55/99 (55.6%) vs. 53/138 (38.4%), chi-square = 6.84, *p* = 0.009]. A larger proportion of participants who were in health-related occupations responded affirmatively [23/36 (63.9%) vs. 76/201 (37.8%), chi-square = 8.54, *p* = 0.003]. The two groups did not differ with respect to Caucasian race [83/99 (83.8%) vs. 117/138 (84.8%), chi-square = 0.039, *p* = 0.84]. In a logistic regression model that included age, gender, Caucasian race, educational level (bachelor’s degree or higher vs. others), and occupation (health-related vs. other), we found that health-related occupation (OR = 2.42, 95% CI = 1.13 – 5.17, *p* = 0.02) and education (OR = 1.96, 95% CI = 1.14 – 3.37, *p* = 0.016) were independently associated with increased odds of responding affirmatively. In PD patients, affirmative response was not associated with mini-mental state screen score (Student’s *t-*test = 0.21, *p* = 0.83).

We asked 99 participants with affirmative responses, the remaining 15 questions concerning the clinical features, disease course, and treatment of ET. Because a similar proportion of general neurology patients, vascular disease patients, and their accompanying persons responded affirmatively to the question about awareness of ET, for the remainder of the analyses, these 74 were combined. The 25 PD patients were still analyzed separately (Table 2). However, as there were few differences between the 25 PD and 74 non-PD participants (Table 2), we combined the data as well (Table 2). More than three-quarters of the participants who responded affirmatively acknowledged that the main symptom of ET was shaky hands (Table 2), yet ~10% could not identify the main symptom and 40% identified the legs as a prominent site of tremor. Nearly all associated the care of ET patients with a neurologist. Approximately 1/3 were either unsure or thought ET was the same disease as PD. Approximately 1/4 thought that ET was the same as frailty- or aging-associated tremor (Table 2). The underlying causes of ET were quite varied, with three-quarters attributing it to genetic inheritance, while similarly high proportions attributed it to odd causes such as trauma or alcohol abuse (Table 2). Nearly 20% thought ET was associated with increased risk of mortality and another 35% were unsure. Nearly two-thirds thought that diet and exercise could help to prevent or control ET. Only one-third knew of the existence of therapeutic brain surgery. Nearly 1/5 thought ET might be curable. The most commonly listed decades of onset were in the 40s, 50s, or 60s, but rarely after that. Less than one-half were aware that children can have ET and nearly 3/4 did not know of a celebrity or historical figure with ET (Table 2).

**Table 2:** **Responses of 99 participants to 15 questions about the clinical features, disease course, and treatment of ET**.

	Participants who did not have PD	Participants who had PD	All participants	Significance (PD vs. non-PD)
*n*	74	25	99	
What are the main symptoms of ET?				*p* = 0.82^a^
Shaky hands	60 (81.1)	19 (76.0)	79 (79.8)	
Other	8 (10.8)	3 (12.0)	11 (11.1)	
Do not know	6 (8.1)	3 (12.0)	9 (9.1)	
What parts of the body can shake when someone has ET?				
Arms (with or without other)	58 (78.4)	8 (32.0)	66 (66.7)	*p* < 0.001^a^
Legs (with or without other)	32 (43.2)	8 (32.0)	40 (40.4)	*p* = 0.45^a^
Cranial structures (with or without other)	34 (45.9)	8 (32.0)	42 (42.4)	*p* = 0.32^a^
Total body	4 (5.4)	7 (28.0)	11 (11.1)	*p* = 0.01^a^
Is ET the same or different from Parkinson’s disease?				*p* = 0.11^a^
The same	10 (13.5)	7 (28.0)	17 (17.2)	
Different	52 (70.3)	17 (68.0)	69 (69.7)	
Do not know	12 (16.2)	1 (4.0)	13 (13.1)	
Is ET the same or different from the type of tremor that many people can get when they become old and frail?				*p* = 0.21^a^
Do not know	12 (16.2)	5 (20.0)	17 (17.2)	
The same	2 (2.7)	3 (12.0)	5 (5.1)	
Similar but not the same	13 (17.6)	2 (8.0)	15 (15.2)	
Different	47 (63.5)	15 (60.0)	62 (62.6)	
What types of doctors generally take care of a patient with ET? Indicate one or more				
General doctor (general practitioner)	17 (23.0)	4 (16.0)	21 (21.2)	*p* = 0.54^a^
Internist	5 (6.8)	2 (8.0)	7 (7.1)	*p* = 0.83^a^
Neurologist	71 (95.9)	25 (100)	96 (97.0)	*p* = 0.31^a^
Neurosurgeon	19 (25.7)	1 (4.0)	20 (20.2)	*p* = 0.02^a^
Not sure	2 (2.7)	0 (0.0)	2 (2.0)	*p* = 0.41^a^
What do you think causes ET? Indicate one or more				
Genes	55 (74.3)	17 (68.0)	72 (72.7)	*p* = 0.54^a^
Trauma	48 (64.9)	14 (56.0)	62 (62.6)	*p* = 0.43^a^
Brain disease	57 (77.0)	20 (80.0)	77 (77.8)	*p* = 0.76^a^
Abnormal dietary habits	22 (29.7)	6 (24.0)	28 (28.3)	*p* = 0.58^a^
Alcohol abuse	46 (62.2)	17 (68.0)	63 (63.6)	*p* = 0.60^a^
Tobacco abuse	28 (37.8)	8 (32.0)	36 (36.4)	*p* = 0.60^a^
Unknown cause	6 (8.1)	1 (4.0)	7 (7.1)	*p* = 0.49^a^
Do you think people with ET die at a younger age than people who do not have ET?				*p* = 0.88^a^
Yes	12 (16.2)	5 (20.0)	17 (17.2)	
No	35 (47.3)	12 (48.0)	47 (47.5)	
Do not know	27 (36.5)	8 (32.0)	35 (35.4)	
Could diet and exercise prevent ET or help to control it?				*p* = 0.41^a^
Yes	44 (59.5)	18 (72.0)	62 (62.6)	
No	18 (24.3)	3 (12.0)	21 (21.2)	
Do not know	12 (16.2)	4 (16.0)	16 (16.2)	
Do you think the symptoms of ET can be medically controlled?				*p* = 0.43^a^
Yes, very well	9 (12.2)	4 (16.0)	13 (13.1)	
Yes, with moderate success	33 (44.6)	13 (52.0)	46 (46.5)	
Yes, but not well	8 (10.8)	0 (0.0)	8 (8.1)	
No	10 (13.5)	2 (8.0)	12 (12.1)	
Do not know	14 (18.9)	6 (24.0)	20 (20.2)	
Is there some type of brain surgery to treat ET?				*p* = 0.58^a^
Yes	21 (28.8)	10 (40.0)	31 (31.3)	
No	18 (24.7)	5 (20.0)	23 (23.2)	
Do not know	34 (46.6)	10 (40.0)	44 (44.4)	
Do you think ET is a curable disease?				*p* = 0.93^a^
Yes	12 (16.7)	5 (20.0)	17 (17.2)	
No	39 (54.2)	13 (52.0)	52 (52.5)	
Do not know	21 (29.2)	7 (28.0)	28 (28.3)	
What is the typical age of onset of ET?				*p* = 0.96^a^
21–30	5 (6.8)	1 (4.0)	6 (6.1)	
31–40	3 (4.1)	2 (8.0)	5 (5.1)	
41–50	8 (10.8)	2 (8.0)	10 (10.1)	
51–60	12 (16.2)	4 (16.0)	16 (16.2)	
61–70	10 (13.5)	4 (16.0)	14 (14.1)	
71–80	3 (4.1)	0 (0.0)	3 (3.0)	
>80	1 (1.4)	0 (0.0)	1 (1.0)	
Any age	24 (32.4)	9 (36.0)	33 (33.3)	
Do not know	8 (10.8)	3 (12.0)	11 (11.1)	
Can children get ET?				*p* = 0.058^a^
Yes	37 (50.0)	8 (32.0)	45 (45.5)	
No	17 (23.0)	12 (48.0)	29 (29.3)	
Do not know	20 (27.0)	5 (20.0)	25 (25.3)	
What do you think is the average memory/thinking deficit of a patient with ET?				*p* = 0.73^a^
No problems with memory/thinking	45 (60.8)	17 (68.0)	62 (62.6)	
Mild problem	18 (24.3)	4 (16.0)	22 (22.2)	
Moderate problem	6 (8.1)	3 (12.0)	9 (9.1)	
Severe problem (dementia)	5 (6.8)	1 (4.0)	6 (6.1)	
Do you know of a celebrity or historical figure with ET				*p* = 0.007^a^
Yes	15 (20.3)	12 (48.0)	27 (27.3)	
No	59 (79.7)	13 (52.0)	72 (72.7)	

*Values are numbers (percentages)*.

*^a^Chi-square test or Fisher’s exact test*.

## Discussion

Essential tremor is estimated to affect seven million individuals in the United States alone (5); indeed, it is one of the most common neurological diseases. Also, it is the most common tremor disorder, as much as 20 times more prevalent than PD (14). However, ET does not have a strong presence in the media and is overshadowed by other movement disorders such as PD. Public awareness as well as public misconceptions of/about disease are important issues for patients (12, 13); however, to our knowledge, these have never been studied in ET.

In the current survey, leaving aside those with PD, only 10–15% of people surveyed had ever heard of or read about ET. In other words, 85–90% had not heard of ET. Even among patients with PD, a related tremor disorder, only 32.7% had ever heard of or read about ET. Providing confirmatory clarification increased these values, but they were still low (~40% of participants without PD and 51.0% participants with PD). By comparison, surveys of epilepsy indicate that more than 90% of respondents are aware of that condition (13).

Even among the minority of participants who had heard of ET, ~10% could not identify its main symptom, ~1/3 could not distinguish ET from PD, ~1/4 thought that ET was the same condition as frailty- or aging-associated tremor, ~2/3 attributed ET to trauma or alcohol abuse, only 1/3 knew of the existence of therapeutic brain surgery, less than one-half knew that children could have ET, and nearly 3/4 did not know of a celebrity or historical figure with ET. Hence, both lack of knowledge and misconceptions were quite common.

Several demographic features (higher educational level and health-related occupation) were associated with greater awareness of ET. To some extent, female gender was associated with greater awareness as well. Yet even among participants in health-related occupations, 13/36 (36.1%) were unaware of ET. Among participants with a bachelor’s degree or higher, 53/108 (49.1%) were unaware of ET.

Parkinson’s disease patients, not surprisingly, were more aware of the existence of ET than were other participants; however, their knowledge of the clinical details was largely similar (Table 2). General neurology patients were no more aware of the existence of ET than were vascular disease patients. Some of this could be due to the fact that the former comprised both established patients as well as new patients who might not have been in a neurological treatment setting of any kind before. Even with this caveat, the lack of difference is intriguing.

Given the lack of public knowledge of ET, one should consider possible interventions for increasing the level of awareness toward ET. Patient-centered foundations already do an excellent job of supporting patient needs and increasing awareness through educational activities; an increase in such activities would be beneficial. Second, a highly visible public spokesperson with ET could also increase awareness more broadly.

This study should be interpreted within the context of several limitations. First, while we surveyed 250 individuals, this number is modest, leaving us with small cells in some of our analyses. In addition, the sample was not strictly population-based, so the results cannot be directly extrapolated to the population. Second, future questionnaires may wish to elicit additional information on the precise sources of information on ET for those participants who were aware of it. This kind of information could guide efforts to extend and increase public knowledge of ET. A strength of the study was the sampling of five different types of individuals who were carefully selected *a priori* to have a range of knowledge of ET, from vascular disease patients who were expected to have the least, to PD patients who were expected to have the most. Second, all individuals who were approached agreed to participate and there were no refusals; by contrast, mailed surveys often have low response rates, making selection bias an issue. Finally, this is the only study to our knowledge to directly address this issue.

In summary, public knowledge of the existence and features of ET is overall poor. Greater awareness is important for the ET community and for organizations that perform public outreach for ET.

## Author Contributions

Research project: conception: SS, EL; organization: SS, EL; execution: SS, JI, BK, CG, DM, AP, and DR. Statistical analysis: design: SS, EL; execution: SS, EL; review and critique: EL. Manuscript: writing of the first draft: SS, EL; review and critique: SS, EL, JI, BK, CG, DM, AP, and DR.

## Conflict of Interest Statement

The authors declare that the research was conducted in the absence of any commercial or financial relationships that could be construed as a potential conflict of interest.
